# Energy intake and dietary fiber as principal determinants of obesity in Eastern Europe, 2010–2022: an ecological panel study

**DOI:** 10.3389/fpubh.2025.1698838

**Published:** 2025-11-10

**Authors:** Rodica Siminiuc, Dinu Țurcanu, Sergiu Siminiuc

**Affiliations:** 1 Faculty of Food Technology, Technical University of Moldova, Chișinău, Moldova; 2 Faculty of Computers, Informatics and Microelectronics, Technical University of Moldova, Chișinău, Moldova

**Keywords:** ecological study, panel data, fixed-effects regression, energy intake, dietary fiber, non-communicable diseases, public health policy

## Abstract

**Background:**

Obesity is a major global health challenge, with Eastern Europe standing out for rapid nutrition transitions and persistent social and economic inequalities. Despite its high prevalence, longitudinal ecological evidence on the structural determinants of obesity in this region remains limited.

**Objective:**

To examine population-level associations between dietary energy availability, dietary fiber intake, macronutrient composition, and insufficient physical activity with obesity and overweight prevalence in Eastern Europe during 2010–2022.

**Methods:**

Data from FAOSTAT and the World Health Organization were assembled into a balanced panel of 130 country–year observations. Analyses combined descriptive statistics and Pearson correlations with two-way fixed-effects regressions (country and year), using robust standard errors and one-year lagged predictors to test for robustness.

**Results:**

Higher energy availability was positively associated with both obesity and overweight, while dietary fiber consistently showed a protective effect. Marginal estimates indicated that an additional 100 kcal/day predicted an increase of nearly one percentage point in obesity, whereas +5 g/day of fiber corresponded to an approximate two-percentage-point reduction. Neither macronutrient shares nor insufficient physical activity showed significant associations.

**Conclusion:**

Dietary energy and fiber emerge as the primary structural correlates of obesity in Eastern Europe. These findings underscore the need for region-specific, data-driven nutrition and public health policies to address obesogenic environments and reduce socio-economic disparities in diet quality.

## 1 Introduction

Overweight and obesity rank among the most urgent global public health concerns, with their prevalence having nearly tripled since 1975, as demonstrated in pooled analyses from the Global Burden of Disease Study (1). This global escalation reflects profound and sustained changes in food environments, energy availability, and physical activity patterns worldwide.

It has been characterized by increased energy availability, reduced fiber intake, and declining levels of physical activity (2, 3). Existing research on the determinants of obesity has predominantly focused on Western countries or global analyses, which limits its applicability to the Eastern European context. In particular, there is a lack of longitudinal evidence covering the post-2010 period, when most Eastern European countries experienced accelerated nutrition transitions and policy diversification.

This analytical gap is particularly relevant for Eastern Europe, where rapid socio-economic transitions, dietary market liberalization, and uneven policy implementation have reshaped dietary and behavioral patterns over a short period (4–8). These shifts have been accompanied by a marked increase in the prevalence of obesity and non-communicable diseases, within a context of pronounced social inequalities and limited resources for prevention (9, 10). Unlike Western Europe, where the nutrition transition was more gradual and supported by public health policies, in Eastern Europe it has been abrupt and insufficiently regulated (7).

The literature on obesity is dominated by individual-level studies—cohort analyses, clinical trials, or population surveys—which provide valuable insights but have limited comparative relevance across countries (1, 11, 12).

Comparative ecological studies remain relatively scarce and are largely focused on global data (13–15) or Western contexts, with limited applicability to Eastern Europe (4, 7, 16). Reports such as those of the FAO (17) or NCD-RisC (18) provide internationally comparable ecological data but are predominantly descriptive and do not employ longitudinal analytical models capable of capturing both cross-country variation and temporal dynamics. Moreover, there is limited quantified evidence on the specific role of dietary factors in this region, particularly dietary fiber, whose protective contribution is recognized but rarely investigated systematically (4, 19, 20).

The objective of this study is to identify and quantify the ecological associations between dietary energy availability, macronutrient composition, dietary fibre, and insufficient physical activity, and adult obesity prevalence across Eastern European countries during 2010–2022, using a two-way fixed-effects panel design.

## 2 Methods

### 2.1 Study design

This study was designed as a panel ecological analysis conducted at the national level, covering ten Eastern European countries: Belarus, Bulgaria, Czechia, Hungary, Poland, the Republic of Moldova, Romania, the Russian Federation, Slovakia, and Ukraine (13). The unit of analysis was the country–year, resulting in a balanced panel dataset with 130 observations. Country selection followed to the FAO/UN M49 regional delineation of Eastern Europe (UN, 2024). This institutional classification, routinely applied in FAO global reports, provides a coherent regional frame for comparative analysis (13, 17).

### 2.2 Data sources

Data on food supply (per capita food availability) were obtained from the FAOSTAT database (Food Balance Sheets, FBS) (15). These datasets provide harmonized national-level estimates of food availability, expressed in kcal/capita/day for total energy and macronutrients, and in g/capita/day for dietary fibre. Epidemiological data on the prevalence of overweight and obesity (defined as BMI ≥ 30 kg/m^2^ and ≥25 kg/m^2^, respectively), as well as the prevalence of insufficient physical activity among adults (≥18 years), were sourced from the World Health Organization Global Health Observatory (21).

These indicators were derived from nationally representative population-based surveys harmonized by WHO. All values were age-standardized (both sexes, adults aged ≥18 years) to the WHO world standard population, ensuring comparability across countries and years (21). Weight and height data were collected through standardized measurement protocols or modelled by WHO when based on self-reported national data, using Bayesian estimation procedures to account for methodological differences and reporting bias. The resulting dataset provides internally consistent and cross-country comparable estimates of population-level overweight, obesity, and physical inactivity.

### 2.3 Variables and definitions

The dietary indicators included total per capita food supply (kcal/capita/day), the proportion of macronutrients in total energy availability (%E for carbohydrates, proteins, and fats), and dietary fibre expressed in original units (g/capita/day). Public health indicators were defined according to the World Health Organization: prevalence of obesity (BMI ≥ 30 kg/m^2^), overweight (BMI ≥ 25 kg/m^2^), and insufficient physical activity (the proportion of adults ≥18 years not meeting WHO recommendations on physical activity). In line with WHO guidelines, insufficient physical activity refers to not achieving 150–300 min/week of moderate-intensity activity or 75–150 min/week of vigorous-intensity activity (or an equivalent combination), including muscle-strengthening activities on at least two days per week (8).

### 2.4 Data processing

Macronutrient values reported by FAOSTAT were converted from kcal/capita/day into percentages of total energy availability, in order to ensure comparability across countries and years, while dietary fibre was retained in its original units (g/capita/day). WHO epidemiological data were used directly in their standardized form. The datasets were merged into a panel file, with each observation corresponding to a country–year. All series were checked for consistency, and no missing or outlier values were identified. To ensure replicability, all time series were visually inspected and tested for internal consistency through year-to-year variation analysis and cross-source comparison (FAOSTAT vs. WHO metadata). Potential outliers were screened using standardized z-scores (|z| > 3) and trend-deviation diagnostics. No extreme or implausible values were detected after validation, and therefore no data imputation or smoothing was applied. Summary statistics and cross-variable consistency checks are reported in the Supplementary Table S1.

### 2.5 Statistical analysis

The statistical analysis included descriptive statistics (means, standard deviations, coefficients of variation) and graphical representations of trends (heatmaps and time series), evaluation of bivariate correlations between dietary factors, physical activity, and weight-related indicators (Pearson coefficients, two-tailed tests), as well as estimation of two-way fixed-effects regression models (country and year) with robust standard errors clustered at the country level. The econometric specification was defined as follows:


$$\begin{array}{l} \text{Obesit}y_{\mathrm{it}}=\beta_{0}+\beta_{1}*\text{Energy}\;100_{\mathrm{it}}+\beta_{2}*\text{Fibre}\;5_{\mathrm{it}}+\\ \beta_{3}\;\mathrm{La}g_{\text{Energy}}100_{i,t}+\beta_{4}*\mathrm{La}g_{\text{Fibre}}5_{i,t-1}+\\ \beta_{5}*\mathrm{IP}A_{\mathrm{it}}+\alpha_{i}+\gamma_{t}+\varepsilon_{\mathrm{it}} \end{array}$$

Where:

Obesity₍_it_₎ – prevalence of obesity for country *i* in year *t*.

*β₀*-constant intercept term.

Energy100₍_it_₎-per capita dietary energy availability, standardized to +100 kcal/day.

Fibre5₍_it_₎-per capita dietary fibre supply, standardized to +5 g/day.

Lag_Energy100₍_i,t–1_₎ and Lag_Fibre5₍_i,t–1_₎—one-year lagged values of dietary energy and fibre, respectively.

IPA₍_it_₎-prevalence of insufficient physical activity among adults.

*α*_i_-country fixed effects, capturing time-invariant unobserved heterogeneity.

*γ*_t_-year fixed effects, controlling for global or regional shocks common across countries.

*ε₍_it_₎*-idiosyncratic error term.

The primary dependent variable was the prevalence of obesity, while overweight prevalence was analyzed exploratorily. Predictors included daily energy availability, macronutrient shares (%E), dietary fibre (g/day), and the prevalence of insufficient physical activity. Due to high collinearity, fats and carbohydrates were tested in separate models. Given the ecological design, all statistical relationships were modelled at the country–year level.

All WHO outcome indicators referred to the adult population (aged 18 years and older) and were age-standardized according to international definitions (WHO Global Health Observatory), ensuring that differences in population age structures across countries and years were already accounted for in the analysis.

For practical interpretation, marginal effects of energy availability and fibre were also expressed on a standardized scale (+100 kcal/day and +5 g/day), while other predictors were interpreted in their original units. Results were reported using *β* coefficients, standard errors, 95% confidence intervals, and *p*-values, with model explanatory power assessed through adjusted R^2^.

Data preprocessing and variable harmonization were conducted in Microsoft Excel with Power Query (Microsoft Corporation, Redmond, WA, United States). Descriptive statistics, Pearson correlations, and graphical trend representations were produced in GraphPad Prism version 10.6.0 (GraphPad Software, San Diego, CA, United States). Two-way fixed-effects panel regressions were estimated in PAST version 5.0 (Øyvind Hammer, Natural History Museum, University of Oslo, Oslo, Norway). The threshold for statistical significance was set at *p* < 0.05.

Robustness checks using one-year lagged predictors (energy availability and dietary fibre) yielded consistent coefficients in sign and magnitude, confirming model stability.

## 3 Results

### 3.1 Descriptive statistics

The descriptive analysis of the ten Eastern European countries during 2010–2022 indicates a relatively homogeneous average daily energy availability, ranging between 3,100 and 3,200 kcal/capita/day. Overall, the regional diet is characterized by carbohydrates providing approximately 48% of total energy, proteins about 12%, and fats around 34%. Dietary fibre availability varied between 30 and 32 g/day, suggesting a relatively uniform pattern across countries (Table 1).

**Table 1:** Descriptive statistics of dietary, physical activity, and weight indicators in 10 Eastern European countries, 2010–2022.

Countries	Energy (kcal/capida/day)		Prevalence of obesity among adults (%)		Prevalence of overweight among adults (%)		Prevalence of insufficient physical activity (IPA) (%)		Carbohydrate (available) supply (E%)		Protein supply (E%)		Fat supply (E%)		Dietary fibre supply (g/d)	
	MEAN ±STDEVP	CV_%	MEAN± STDEVP	CV_%	MEAN	CV_%	MEAN	CV_%	MEAN	CV_%	MEAN	CV_%	MEAN	CV_%	MEAN	CV_%
Belarus	3.197 ± 234	7.3	21.1 ± 0.2	0.8	54.3 ± 0.7	1.3	15.1 ± 0.7	4.9	47.8 ± 3.9	8.2	12.3 ± 0.9	7.2	34.0 ± 4.1	12.1	32 ± 6	19.6
Bulgaria	3.183 ± 200	6.3	18.8 ± 1.1	5.6	50.8 ± 0.7	1.5	28.6 ± 2.2	7.8	47.8 ± 4.3	9.0	12.3 ± 1.0	8.1	34.1 ± 4.7	13.7	31 ± 6	19.7
Czechia	3.211 ± 200	6.2	23.4 ± 1.4	6.2	57.3 ± 1.3	2.3	23.2 ± 0.1	0.3	48.3 ± 4.8	9.9	12.4 ± 0.9	7.3	33.6 ± 4.9	14.7	32 ± 6	19.3
Hungary	3.205 ± 235	7.3	27.7 ± 2.3	8.3	59.4 ± 1.7	2.8	25.9 ± 2.1	8.2	48.5 ± 4.9	10.1	12.3 ± 0.8	6.8	33.6 ± 5.2	15.6	32 ± 6	20.0
Poland	3.171 ± 243	7.7	24.3 ± 1.9	7.7	59.4 ± 2.1	3.5	32.5 ± 2.7	8.3	47.9 ± 5.1	10.7	12.1 ± 0.8	6.2	34.3 ± 5.3	15.4	31 ± 7	21.2
Republic of Moldova	3.094 ± 247	8.0	23.0 ± 0.1	0.3	58.6 ± 1.6	2.7	12.0 ± 0.8	6.8	47.9 ± 5.1	10.7	12.1 ± 0.7	5.9	34.5 ± 5.4	15.5	30 ± 6	20.4
Romania	3.119 ± 251	8.0	26.7 ± 4.3	16.1	59.0 ± 3.7	6.2	31.0 ± 3.5	11.3	47.9 ± 5.7	12.0	12.0 ± 0.8	6.4	34.3 ± 5.8	17.0	30 ± 7	21.4
Russian Federation	3.139 ± 247	7.9	23.0 ± 0.7	3.0	56.2 ± 1.6	2.8	15.7 ± 1.4	9.2	48.9 ± 5.5	11.2	12.1 ± 0.6	5.2	33.1 ± 5.2	15.6	31 ± 5	17.4
Slovakia	3.187 ± 254	8.0	23.1 ± 2.1	9.2	57.6 ± 1.8	3.1	23.9 ± 0.5	2.0	48.6 ± 5.0	10.3	12.2 ± 0.6	5.3	33.3 ± 4.8	14.5	32 ± 6	19.1
Ukraine	3.165 ± 267	8.4	22.5 ± 0.7	2.9	53.8 ± 1.3	2.5	12.0 ± 0.5	3.9	48.5 ± 4.5	9.2	12.2 ± 0.9	7.1	33.5 ± 4.8	14.3	31 ± 6	19.5

The table presents mean, STDEVP, and coefficient of variation (CV%) for daily energy intake (kcal/capita), macronutrient composition (%E), dietary fibre (g/day), prevalence of obesity (BMI ≥30), overweight (BMI ≥25), and insufficient physical activity (% adults 18+). Values are shown overall and per country.

Prevalence of obesity (BMI ≥ 30) showed marked differences across countries. The lowest values were recorded in Bulgaria (18.8%), while the highest were observed in Hungary (27.7%) and Romania (26.7%), both exceeding the Eastern European average (Supplementary Figure S1).

A similar trend was noted for overweight (BMI ≥ 25), with the highest prevalence in Poland and Hungary (59.4%), followed by Romania (59.0%) and Moldova (58.6%), and the lowest in Bulgaria (50.8%). The highest level of physical inactivity was observed in Poland (32.5%), whereas the lowest was found in both Moldova and Ukraine (12.0%) (Supplementary Figure S2).

### 3.2 Correlations between main variables

**Figure 1:**
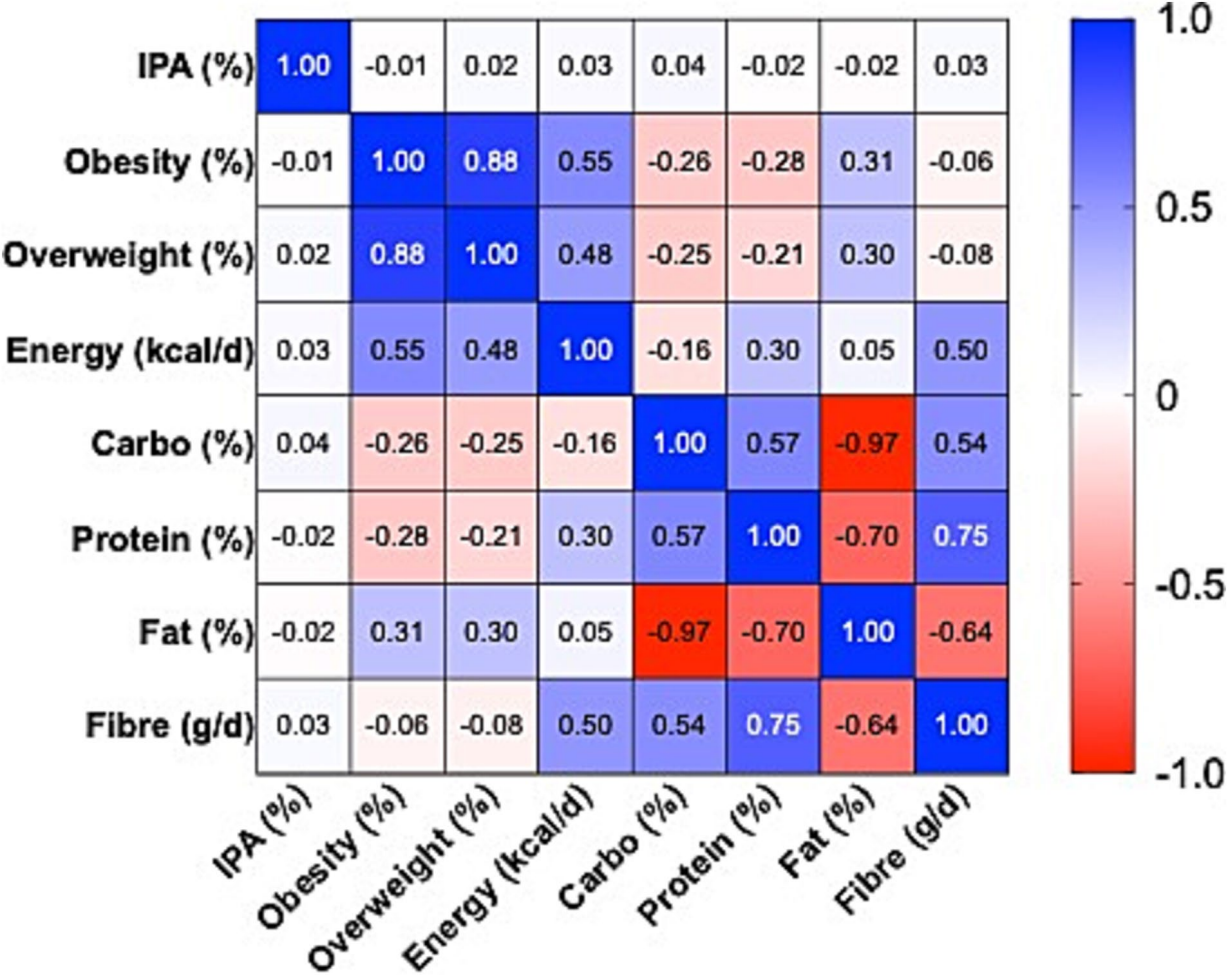
Heatmap of Pearson correlations between dietary factors, physical inactivity, and prevalence of obesity and overweight, Eastern Europe, 2010–2022. Pearson correlation coefficients (r) displayed as a heatmap with scale −1 (blue) to +1 (red), 0 = white. Values printed in cells (2 decimals). Variables: daily energy availability, fat %E, carbohydrate %E, protein %E, fibre (g/day), insufficient physical activity (%), obesity and overweight prevalence. Based on 130 country–year observations.

The bivariate correlation analysis (Figure 1; Supplementary Table S1) revealed a very strong association between obesity and overweight prevalence (r = 0.88), confirming the epidemiological consistency of the two indicators. Daily energy availability was positively correlated with obesity (r = 0.55) and overweight (r = 0.48), while dietary fibre showed weak and non-significant negative correlations (r = −0.06 and r = −0.08, respectively).

Regarding macronutrients, fat availability was positively correlated with obesity (r = 0.31), whereas carbohydrates and proteins were negatively correlated (r = −0.26 and r = −0.28, respectively), all statistically significant. Structurally, extreme correlations were observed among macronutrients: carbohydrates and fats displayed an almost perfect inverse relationship (r = −0.97), while proteins and fibre exhibited a strong positive association (r = 0.75).

In contrast, the prevalence of insufficient physical activity did not show meaningful correlations with either obesity (r = −0.007) or overweight (r = 0.025), with confidence intervals including zero. This finding suggests that cross-country and inter-annual variability in insufficient physical activity does not directly explain differences in excess weight prevalence, compared with dietary factors, which displayed more consistent associations.

Nonetheless, they provide an important foundation for subsequent multivariate models and underscore in particular the role of energy availability and macronutrient composition in relation to obesity and overweight in Eastern Europe. Based on these results, fixed-effects regression models were estimated to test the robustness of the associations by accounting for unmeasured country characteristics and common annual variation.

### 3.3 Fixed-effects regression models for obesity

The fixed-effects analyses revealed a consistent association between energy availability and obesity prevalence, with coefficients positive and statistically significant in both model specifications (*β* = 0.0101 and β = 0.0106; 95% CI: 0.0079–0.0128; *p* < 0.001). All estimates are ecological and associational; they reflect relationships at the country–year level and cannot be interpreted as causal effects. Dietary fibre demonstrated a robust inverse association, with coefficients negative and significant (*β* ranging from −0.41 to −0.44; *p* < 0.001), and confidence intervals entirely below zero (Table 2).

**Table 2:** Fixed-effects regression results for adult obesity prevalence in Eastern Europe, 2010–2022 (specifications including Fat %E or Carbohydrate %E).

Predictor	β (Fat%E)	SE	IC95% (inf–sup)	*p*	β (Carbo%E)	SE	IC95% (inf–sup)	*p*
Energy (kcal/zi)	0.0101	0.0011	0.0079–0.0123	<0.001	0.0106	0.0011	0.0084–0.0128	<0.001
Fat (%E)	0.0064	0.0689	−0.130 – 0.143	0.926	–	–	–	–
Carbohydrate (%E)	–	–	–	–	0.0416	0.0710	−0.099 – 0.182	0.559
Fibre (g/zi)	−0.4060	0.0677	−0.540 – −0.272	<0.001	−0.4370	0.0608	−0.558 – −0.316	<0.001
IPA (%)	0.0089	0.0857	−0.161 – 0.179	0.918	0.0128	0.0855	−0.156 – 0.182	0.882

**Figure 2:**
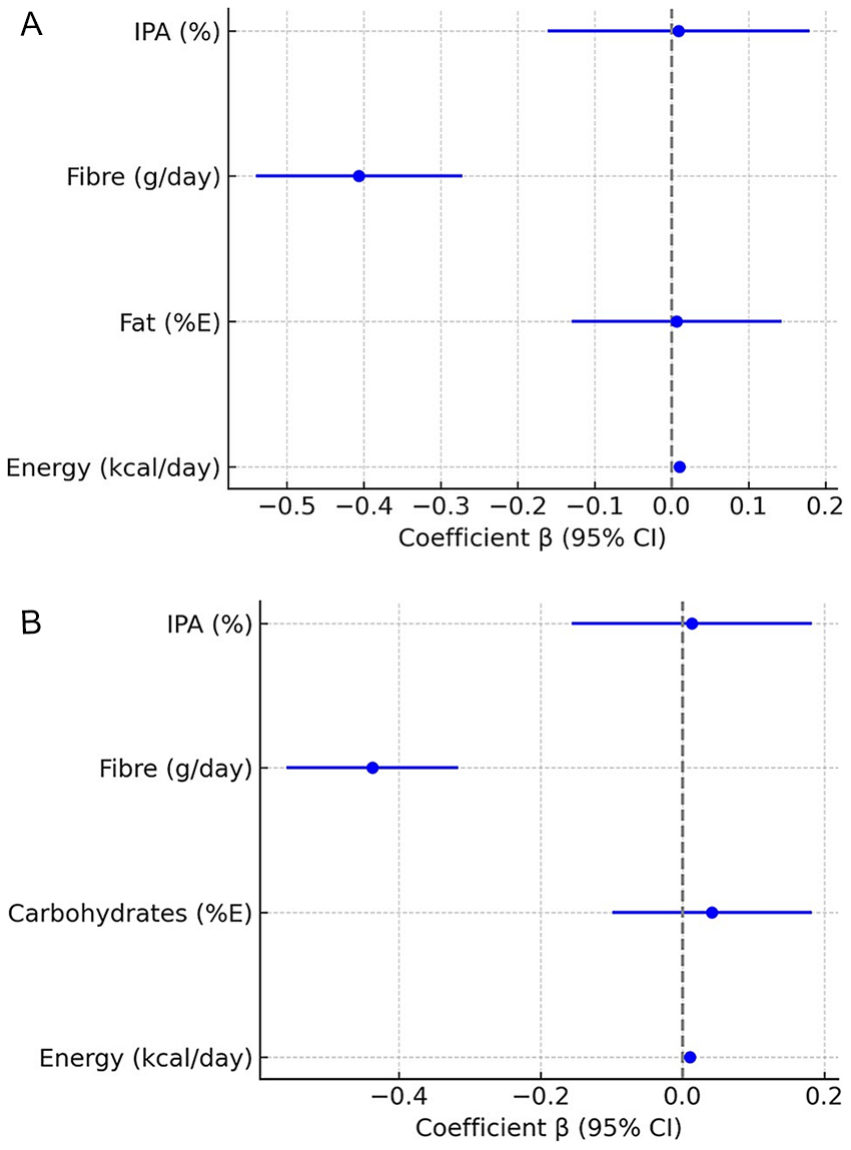
**(A)** Associations between dietary factors and adult obesity prevalence – FE model with fat (%E). Forest plot of *β* coefficients (points) with 95% confidence intervals (horizontal bars) from fixed-effects regressions. Dependent variable: adult obesity prevalence (%). Predictors: energy availability, fat %E, fibre intake, insufficient physical activity. Country and year dummies included but not shown. *N* = 130; adjusted R^2^ = 0.787. Vertical line marks β = 0; bars not crossing 0 indicate statistical significance. **(B)** Associations between dietary factors and adult obesity prevalence – FE model with carbohydrate (%E). Forest plot of β coefficients with 95% CI from FE regressions. Dependent variable: adult obesity prevalence (%). Predictors: energy availability, carbohydrate %E, fibre intake, insufficient physical activity. Country/year dummies included but not shown. *N* = 130; adjusted R^2^ = 0.788. Vertical line marks β = 0.

Regression coefficients (β), standard errors (SE), 95% confidence intervals (CI), and *p*-values are reported for two model specifications. The first specification includes total energy intake, fat (% of energy), fibre intake (g/day), and insufficient physical activity (%). The second specification replaces fat with carbohydrate (% of energy), while keeping the other predictors identical. Both specifications were estimated with country- and year-fixed effects (coefficients not shown). Adjusted R^2^ = 0.787 and 0.788, respectively. F(25,104) = 20.1 and 20.2, *p* < 0.001. *N* = 130 observations. Coefficients in Table 2 and Figures 2A,B are reported per 1 kcal and per 1 g of fiber.

In the specification including the proportion of fat (%E), this predictor was not significant (*p* = 0.93), nor was insufficient physical activity (*p* = 0.92) (Figure 2A). In the specification including the proportion of carbohydrates (%E), the results were similar: carbohydrates showed no significant effects (*p* = 0.56), and insufficient physical activity remained non-significant (*p* = 0.88) (Figure 2B).

### 3.4 Marginal predictions and robustness checks

The analysis of marginal contrasts indicated that total energy availability and dietary fibre show the strongest ecological associations with obesity and overweight prevalence in Eastern Europe between 2010 and 2022. For obesity, an increase of 100 kcal per capita per day was associated with a 0.9–percentage point rise in prevalence (*β* = 0.0089, 95% CI: 0.0063–0.0115, *p* < 0.001), whereas an additional 5 g/day of fibre was associated with a 2.1–percentage point reduction (*β* = −0.205, 95% CI: −0.28 to −0.13, *p* < 0.001).

**Figure 3:**
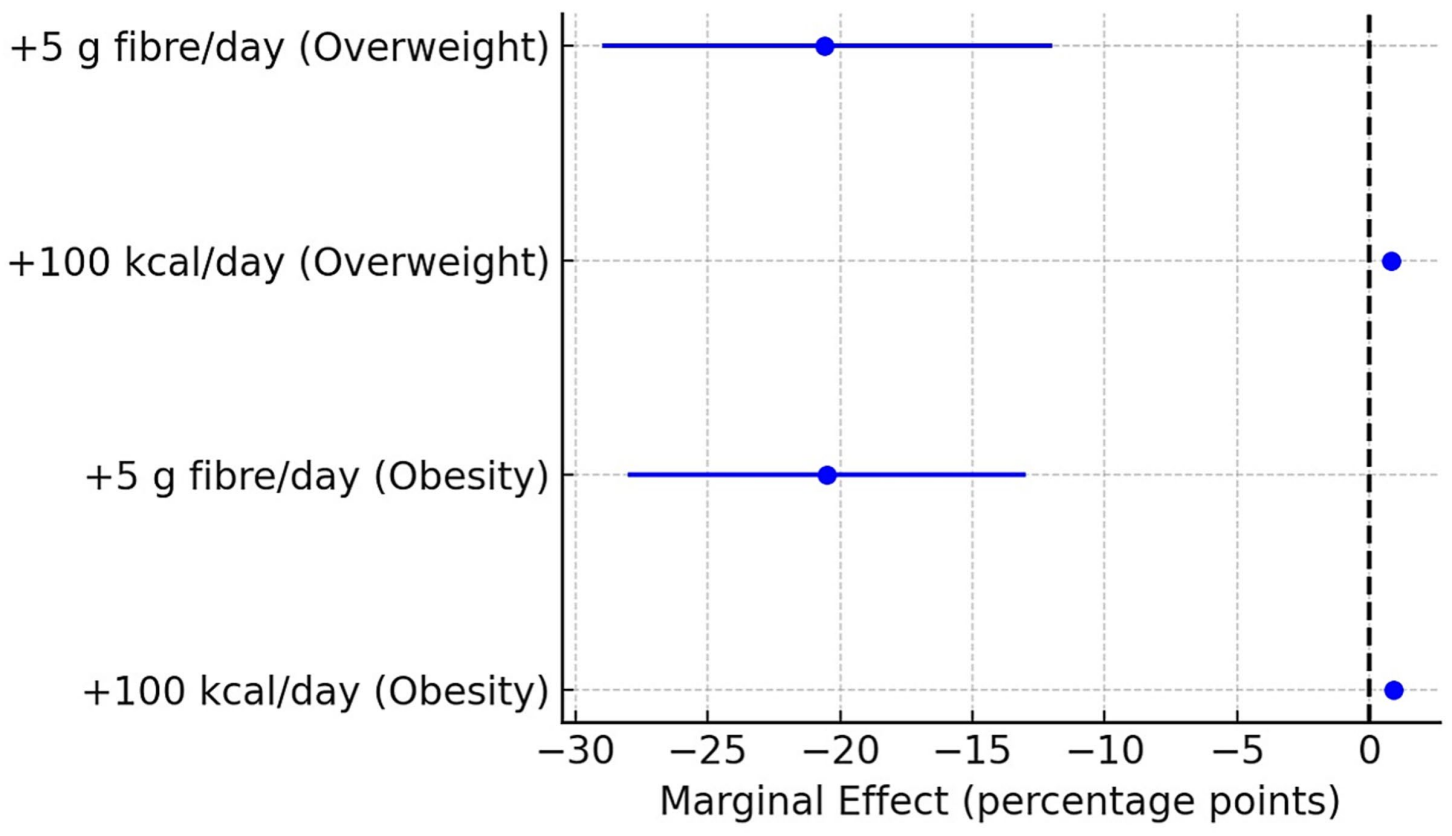
Marginal effects of dietary energy availability (+100 kcal/day) and fibre (+5 g/day) on obesity and overweight prevalence in Eastern Europe, 2010–2022. The points represent estimated marginal effects (percentage points) from two-way fixed-effects panel regressions (country and year effects controlled). Horizontal lines indicate 95% confidence intervals. Positive values indicate an increased prevalence associated with the dietary change, while negative values indicate a protective effect. Energy availability was modelled per +100 kcal/day and fibre intake per +5 g/day. Results are shown separately for obesity and overweight prevalence. For more intuitive interpretation, predictors were rescaled: energy to +100 kcal/day, fibre to +5 g/day, and insufficient physical activity to +5 percentage points.

Similar magnitude and direction of effects were observed for overweight, with a 0.8–percentage point increase per 100 kcal (β = 0.0084, 95% CI: 0.0063–0.0105, *p* < 0.001) and a 2.1–percentage point reduction per 5 g of fibre (β = −0.206, 95% CI: −0.29 to −0.12, *p* < 0.001) (Figure 3; Supplementary Table S2).

Physical inactivity and the macronutrient composition expressed as a percentage of total energy availability did not show significant marginal effects in the main models. Complete results for these predictors are provided in the Supplementary Figure S3.

### 3.5 Exploratory analyses (overweight and robustness)

The results for overweight confirmed the pattern observed for obesity. Daily energy intake showed a positive and significant association (*β* = 0.0083; *p* < 0.001), corresponding to an increase of approximately 0.8 percentage points in overweight prevalence for each additional 100 kcal. Dietary fibre demonstrated a robust protective effect (*β* ranging from −0.27 to −0.31; *p* < 0.001), equivalent to a reduction of about 2 percentage points for every additional 5 g/day. In contrast, insufficient physical activity was not significantly associated with overweight (*p* > 0.70).

Additional analyses of macronutrients revealed modest and inconsistent effects: a positive coefficient for fat (%E) (β = 0.19; *p* = 0.022) and a negative coefficient for carbohydrates (%E) (β = −0.17; *p* = 0.044). However, these findings do not alter the main direction of associations for energy and fiber. Given the ecological design and the strong intercorrelations between dietary variables, these exploratory associations should be interpreted cautiously.

The explanatory power of the fixed-effects models remained high (adjusted R^2^ = 0.75), confirming the robustness of the results. Graphical representations and alternative specifications are presented in the Supplementary Table S3 and Supplementary Figures S4a,b.

## 4 Discussion

This study makes several methodological and conceptual contributions. It applies a two-way fixed-effects ecological panel design controlling for country and year heterogeneity, thereby enabling robust estimation of regional diet–obesity associations. The integration of harmonized FAOSTAT and WHO indicators into a reproducible longitudinal framework demonstrates that openly available data can yield meaningful population-level insights when systematically analysed (22). The use of standardized marginal effects further improves interpretability for policymakers by expressing associations in percentage-point changes. Together, these features position the study as a reproducible framework for regional nutrition and obesity surveillance in Eastern Europe.

### 4.1 Positioning the results in the global context

The findings of this study, highlighting the central role of energy availability and dietary fibre in the rising prevalence of obesity and overweight, are consistent with global trends reported by the World Health Organization and FAO. Annual FAO reports (17) and analyses by the NCD Risk Factor Collaboration (1), show that the rapid increase in obesity is correlated with excessive energy availability and declining consumption of fibre-rich foods (23). These sources confirm that the global obesity epidemic is strongly driven by the imbalance between available energy and the intake of protective nutrients.

However, international reports remain predominantly descriptive and do not capture in detail how these factors vary across Eastern European countries. Within this framework, the study delivers robust quantitative estimates that allow for a clearer evaluation of the magnitude of dietary effects on obesity at the regional level. This approach offers a more nuanced picture of the nutrition transition, demonstrating that beyond global trends, important regional specificities exist and deserve separate investigation.

In this regard, the study complements FAO and WHO reports by focusing specifically on Eastern Europe and providing strong statistical evidence of the link between food availability and obesity prevalence. This comparative and longitudinal framework highlights both convergence with global patterns and distinctive regional features, thereby adding value to the existing literature and opening perspectives for public health policies tailored to the Eastern European context.

### 4.2 The specificity of Eastern Europe in the nutrition transition

Eastern Europe provides a distinctive context for analyzing the determinants of obesity, differing from that of Western Europe and other developed regions. After the 1990s, market liberalization and profound socio-economic changes triggered a rapid shift from traditional dietary patterns, based on whole grains, legumes, and locally produced plant foods, toward an increased availability of processed, energy-dense products. Unlike Western Europe, where the nutrition transition was gradual and accompanied by public health policies, in Eastern Europe these changes occurred over a short period and with limited compensatory measures (6, 22, 24).

Across the European Union, compensatory measures to improve population diets have included front-of-pack nutrition labelling, nutrition standards for school meals and public procurement, restrictions on the marketing of unhealthy foods to children, and product reformulation targets. Implementation has varied substantially by country in terms of timing, scope, and enforcement, including in Eastern Europe (25–28).

This dynamic partly explains why some countries in the region, such as Romania and Hungary, experienced steep increases in obesity, correlated with high caloric availability. By contrast, in countries such as the Republic of Moldova or Ukraine, although caloric levels were comparable, obesity prevalence rose more slowly, suggesting a more important role of dietary structure and the accessibility of processed foods. In Poland and Czechia, obesity trends appear to have been influenced by additional factors, including agricultural and trade policies that shaped access to specific food categories (29, 30).

Thus, the socio-economic and institutional vulnerabilities specific to Eastern Europe not only accompany the nutrition transition but also amplify the impact of dietary factors, explaining why obesity trends have been faster and more heterogeneous than in other European regions. These observed trendlines align with the expected direction of nutritional transition indicators, showing that rising obesity coincides with higher caloric availability and declining dietary fibre. The convergence between these empirical results and well-documented dietary shifts in the region (6, 22, 31) reinforces the internal validity of the analysis and indicates that the dataset reflects genuine structural patterns rather than measurement inconsistencies.

### 4.3 Scientific contribution and methodological value

Unlike descriptive global reports, this study applies a two-way fixed-effects design that controls for country and year effects, producing robust regional estimates of the relationship between food availability and obesity (18). The high explanatory power of the models (adjusted R^2^ = 0.78) confirms that this approach captures substantial regional variation in obesity, a feature rarely achieved in ecological studies. A scoping search of the literature identified few studies directly comparable with this design: most are global panel analyses or country-specific cross-sectional accounts, and where panel approaches exist, they rarely focus on Eastern Europe and differ in indicator definitions, age groups, and time frames (6, 22, 32–34).

An additional contribution lies in the use of standardized marginal effects, which translate model coefficients into directly interpretable units for public policy (35). This step transforms the results from mere statistical associations into operational information readily applicable in the formulation of nutrition policies. Moreover, the introduction of lagged variables allowed for robustness testing and the evaluation of potential delayed effects, further strengthening the validity of the conclusions.

Thus, the study goes beyond descriptive analysis and provides a reproducible analytical framework, demonstrating that FAOSTAT and WHO data can yield relevant insights when integrated into a longitudinal design and interpreted with robust statistical tools.

### 4.4 Limitations and future research directions

This study has several limitations inherent to its ecological design and the data sources used. First, FAOSTAT Food Balance Sheet data reflect per capita food availability rather than actual individual consumption, which may introduce discrepancies between available and effectively consumed foods. Second, WHO data on physical activity are population-level estimates and may obscure important variations across socio-demographic groups or subregions. This characteristic may explain the lack of significant ecological associations and suggests caution when interpreting results related to insufficient physical activity. Third, the ecological design does not allow for causal inference at the individual level and does not capture finer behavioral interactions, such as the combined effects of diet and physical activity.

Additionally, although harmonized international data minimize systematic bias, the possibility of residual measurement error cannot be entirely excluded. The applied two-way fixed-effects regression was considered appropriate to control for unobserved heterogeneity across countries and years, supporting the robustness of the estimates. Importantly, the associations identified in this study reflect population-level risks rather than individual-level relationships, consistent with the ecological nature of the analysis.

Nevertheless, these limitations do not invalidate the conclusions but instead define the framework within which they should be interpreted. A major strength of the study lies in the use of harmonized and internationally comparable data sources, integrated into a longitudinal panel design with fixed effects, which provides a robust perspective on the structural determinants of obesity in the region. In addition, the results highlight consistent patterns and associations that can inform hypotheses for more detailed individual-level studies.

Future research should include: (i) longitudinal individual-level studies linking dietary patterns with weight outcomes; (ii) mixed analyses combining ecological and individual data for a more comprehensive understanding; (iii) evaluation of the impact of food and fiscal policies on obesity prevalence in Eastern Europe; and (iv) extension of the comparative approach to other regions undergoing nutrition transition, such as the Caucasus or Central Asia; (v) multi-country panel studies using harmonised indicator definitions and aligned time coverage—ideally including subnational series—to improve cross-country comparability and test the generalisability of our findings; (vi) cross-country quasi-experimental evaluations (e.g., difference-in-differences or synthetic control) of major nutrition policies to strengthen causal inference where feasible.

Overall, while this study cannot capture individual-level mechanisms of weight gain, it provides a solid empirical foundation for future research and for developing evidence-based public health policies adapted to the socio-economic and dietary context of Eastern Europe.

## 5 Conclusion

The aim of this study was to evaluate the structural factors associated with the evolution of obesity in Eastern Europe, using harmonised data and a longitudinal analytical framework. The analysis confirms that ecological approaches can generate robust and comparable evidence, capable of capturing regional specificities and complementing existing global perspectives. Overall, understanding and controlling obesity in this region requires data-driven public policies adapted to the local context and addressing the identified determinants in a systemic and co-ordinated manner.

## Data Availability

Publicly available datasets were analyzed in this study. This data can be found here: FAOSTAT—Food Balance Sheets: https://www.fao.org/faostat/en/#data/FBS; WHO—Global Health Observatory (GHO) https://www.who.int/data/gho.
