# Supplementary material for: Energy intake and dietary fiber as principal determinants of obesity in Eastern Europe, 2010–2022: an ecological panel study
Source: Front Public Health. 2025 Nov 10;13:1698838. doi: 10.3389/fpubh.2025.1698838 (PMC12640812; doi:10.3389/fpubh.2025.1698838)
Supplement: Supplementary file 1 [file Data_Sheet_1.docx]

Supplementary Material

# Supplementary Tables

**Table S1: Pearson correlation matrix between dietary indicators, physical inactivity, and prevalence of obesity and overweight, Eastern Europe, 2010–2022.**

|  | **IPA (%)** | **Obesity (%)** | **Overweight (%)** | **Energy (kcal/d)** | **Carbo (%)** | **Protein (%)** | **Fat (%)** | **Fibre (g/d)** |
| --- | --- | --- | --- | --- | --- | --- | --- | --- |
| **IPA (%)** | 1 | -0,006903544 | 0,024571301 | 0,028487571 | 0,037939568 | -0,015190446 | -0,016661874 | 0,025046541 |
| **Obesity (%)** | -0,006903544 | 1 | 0,879166506 | 0,553479155 | -0,258635716 | -0,275954251 | 0,308221051 | -0,055492628 |
| **Overweight (%)** | 0,024571301 | 0,879166506 | 1 | 0,476824869 | -0,254546289 | -0,207510601 | 0,301908041 | -0,076064253 |
| **Energy (kcal/d)** | 0,028487571 | 0,553479155 | 0,476824869 | 1 | -0,155487128 | 0,303072756 | 0,052938728 | 0,50385028 |
| **Carbo (%)** | 0,037939568 | -0,258635716 | -0,254546289 | -0,155487128 | 1 | 0,571557763 | -0,973847521 | 0,540122473 |
| **Protein (%)** | -0,015190446 | -0,275954251 | -0,207510601 | 0,303072756 | 0,571557763 | 1 | -0,699700919 | 0,748597198 |
| **Fat (%)** | -0,016661874 | 0,308221051 | 0,301908041 | 0,052938728 | -0,973847521 | -0,699700919 | 1 | -0,642785322 |
| **Fibre (g/d)** | 0,025046541 | -0,055492628 | -0,076064253 | 0,50385028 | 0,540122473 | 0,748597198 | -0,642785322 | 1 |
| **Legend:** The table reports Pearson correlation coefficients (r) between dietary composition (%E macronutrients, fibre intake), daily energy intake, insufficient physical activity, and prevalence of obesity and overweight, based on country–year observations (n=130). P-values were calculated with two-tailed tests. | | | | | | | | |

**Table S2. Associations between diet, physical activity, and the prevalence of obesity and overweight (fixed-effects models with lag variables, 2010–2022, N=130)**

| **Predictor** | **β Obesity (95% CI)** | **p-value** | **β Overweight (95% CI)** | **p-value** |
| --- | --- | --- | --- | --- |
| Energy (kcal/100) | 0.0089 (0.0063 – 0.0115) | <0.001 | 0.0084 (0.0063 – 0.0105) | <0.001 |
| Fibre (g/5) | −0.205 (−0.28 – −0.13) | <0.001 | −0.206 (−0.29 – −0.12) | <0.001 |
| Lag Energy | 0.0025 (0.0006 – 0.0044) | 0.012 | 0.0033 (0.0011 – 0.0055) | 0.004 |
| Lag Fibre | −0.099 (−0.18 – −0.02) | 0.020 | −0.087 (−0.18 – 0.01) | 0.075 |
| Physical activity (%) | −0.010 (−0.06 – 0.04) | 0.708 | 0.003 (−0.06 – 0.07) | 0.921 |
| **Legend:** Fixed-effects panel regression models (country and year effects controlled) were used to estimate the associations between dietary factors, physical activity, and the prevalence of obesity and overweight. Independent variables included total energy intake (per 100 kcal), fibre intake (per 5 g), lagged energy (1-year delay), lagged fibre (1-year delay), and prevalence of insufficient physical activity (%). Regression coefficients (β) are presented with 95% confidence intervals (CI) and p-values. **Note:**Coefficients for country and year dummies (fixed effects) and the constant are not reported. | | | | |

**Table S3.** **Fixed-effects regression results for adult overweight prevalence in Eastern Europe, 2010–2022 (specifications including Fat %E or Carbohydrate %E).**

| **Predictor** | **β (Fat%E)** | **SE** | **IC95% (inf–sup)** | **p** | **β (Carbo%E)** | **SE** | **IC95% (inf–sup)** | **p** | |
| --- | --- | --- | --- | --- | --- | --- | --- | --- | --- |
| Energy (kcal/zi) | 0.00824 | 0.00127 | 0.0057 – 0.0108 | <0.001 | 0.00838 | 0.00131 | 0.0058 – 0.0110 | <0.001 | |
| Fat (%E) | 0.1854 | 0.0796 | 0.028 – 0.343 | 0.022 | – | – | – | – | |
| Carbohydrate (%E) | – | – | – | – | −0.169 | 0.0826 | −0.332 – −0.005 | 0.044 | |
| Fibre (g/zi) | −0.270 | 0.0783 | −0.425 – −0.115 | <0.001 | −0.308 | 0.0708 | −0.448 – −0.167 | <0.001 | |
| IPA (%) | 0.0282 | 0.0991 | −0.168 – 0.225 | 0.777 | 0.0348 | 0.0995 | −0.161 – 0.231 | 0.727 | |
| **Legend:** Regression coefficients (β), standard errors (SE), 95% confidence intervals (CI), and p-values are reported for two specifications. The first includes total energy intake, fat (% of energy), fibre intake (g/day), and insufficient physical activity (%). The second replaces fat with carbohydrate (% of energy), keeping the other predictors constant. Both specifications include country- and year-fixed effects (coefficients not shown). Adjusted R² = 0.751 and 0.748, respectively. F(25,104) = 16.5 and 16.3, p < 0.001. N = 130 observations. | | | | | | | | |  |


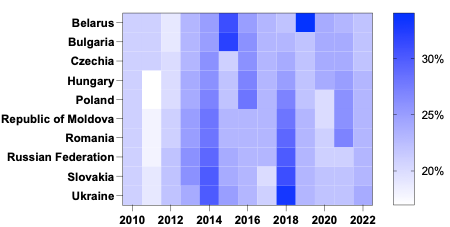


**Figure S1. Trends in adult obesity prevalence by country, Eastern Europe, 2010–2022.
Legend:** Heatmap showing annual prevalence of adult obesity (BMI ≥30) across 10 Eastern European countries. Colours represent percentage prevalence (scale 0–35%). Data from WHO Global Health Observatory.


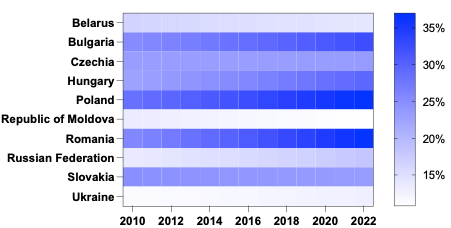


**Figure S2. Trends in prevalence insufficient physical activity among adults, Eastern Europe, 2010–2022.**

**Legend:** Heatmap of adult insufficient physical activity prevalence (% of population aged 18+ not meeting WHO recommendations) across 10 Eastern European countries, 2010–2022. Scale consistent with Figure 1.

**
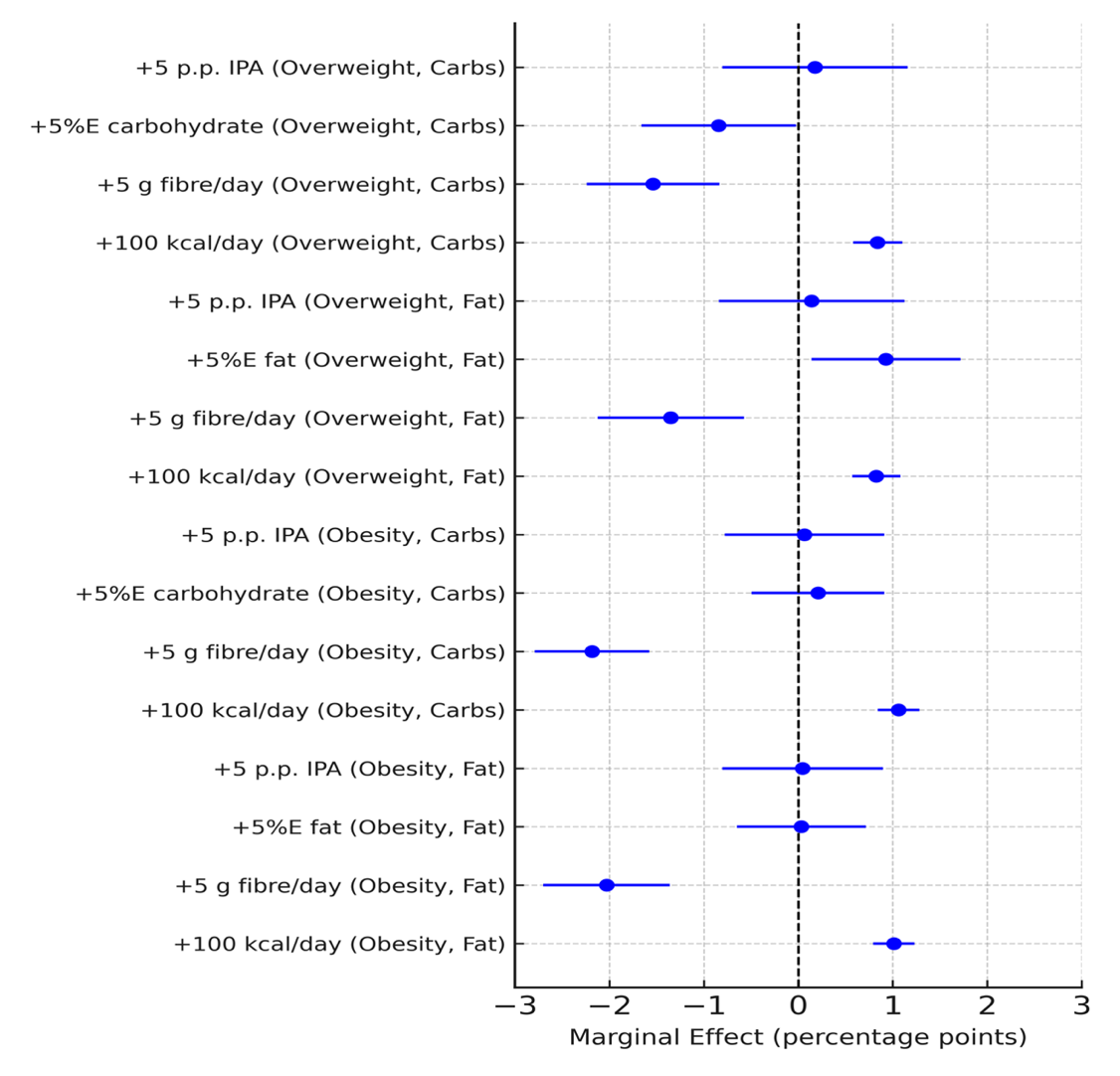
**

**Figure S3. Forest/ME. Marginal effects of dietary predictors on obesity and overweight in Eastern Europe (2010–2022).**

**Legend:** The points represent the estimated marginal effects (percentage points) from fixed-effects panel regressions. Horizontal lines indicate 95% confidence intervals. Positive values reflect increased prevalence associated with the dietary change, while negative values indicate a protective effect. Energy intake was modeled per +100 kcal/day, fibre per +5 g/day, macronutrients per +5% of total energy (%E), and Insufficient Physical Activity (IPA) per +5 percentage points. Results are shown separately for obesity and overweight, with specifications including either fat %E or carbohydrate %E.

| ~~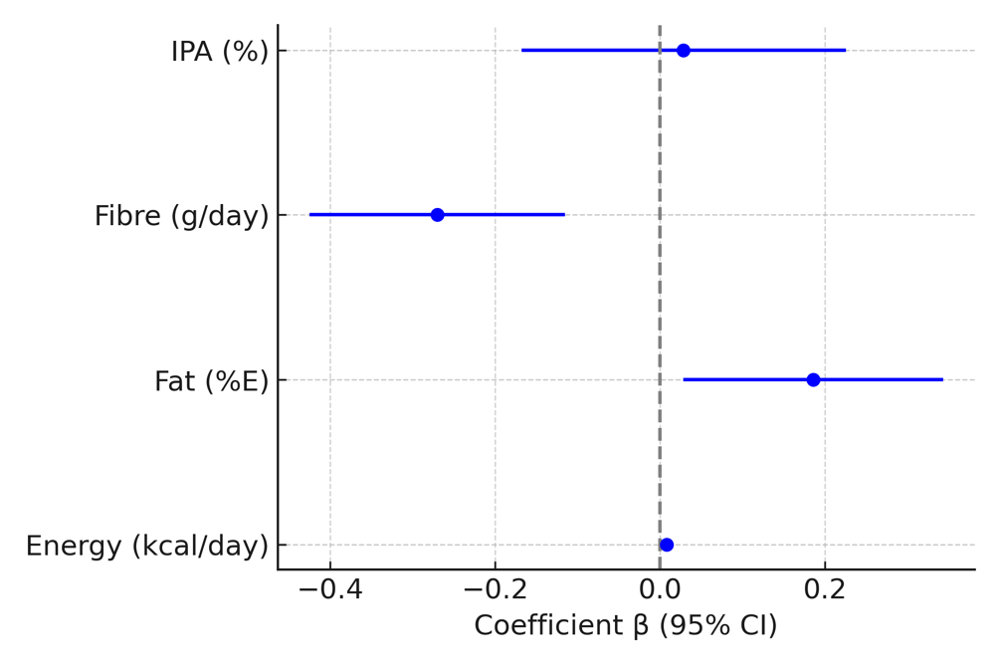~~ | ~~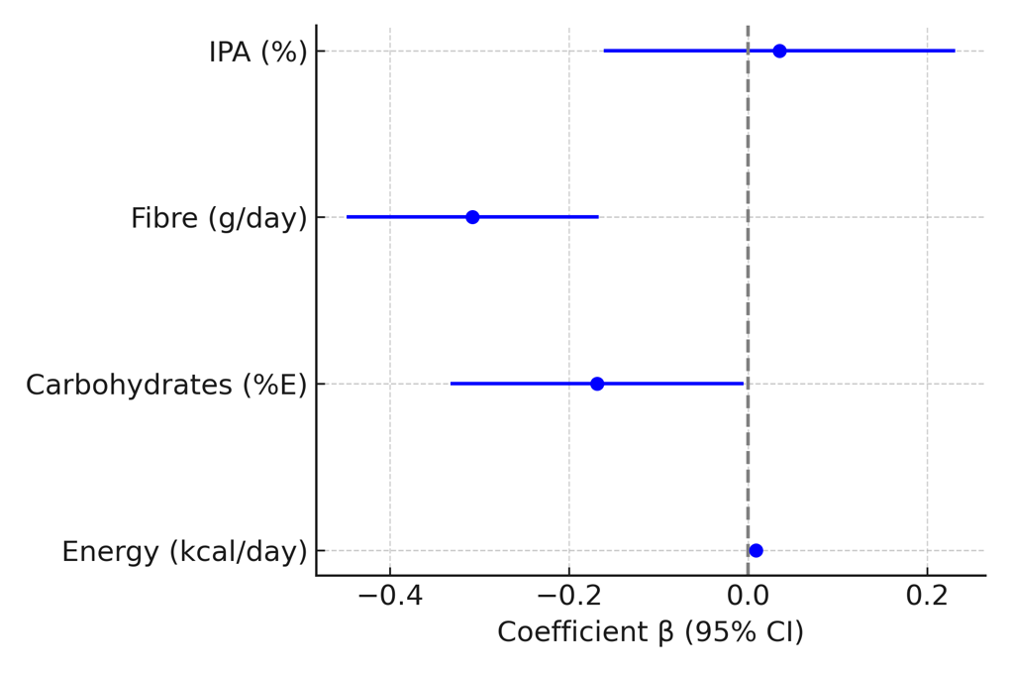~~ |
| --- | --- |
| **Figure S4a. Forest/Me. Associations between dietary factors and adult overweight prevalence – FE model with fat (%E).**  **Legend:** Forest plot of β coefficients with 95% CI from FE regressions. Dependent variable: adult overweight prevalence (%). Predictors: energy intake, fat %E, fibre intake, insufficient physical activity. Country/year dummies included but not shown. N=130; adjusted R²=0.751. | **Figure S4b. Forest/Me. Associations between dietary factors and adult overweight prevalence – FE model with carbohydrate (%E).** **Legend:** Forest plot of β coefficients with 95% CI from FE regressions. Dependent variable: adult overweight prevalence (%). Predictors: energy intake, carbohydrate %E, fibre intake, insufficient physical activity. Country/year dummies included but not shown. N=130; adjusted R²=0.748. |
